# The value of enhanced multiparameteric MRI diagnostic model for preoperatively predicting surgical methods of inferior vena cava in patients with renal tumors and inferior vena cava tumor thrombus

**DOI:** 10.1186/s12880-023-01043-0

**Published:** 2023-06-24

**Authors:** Xinlong Pei, Min Lu, Zhuo Liu, Baohua Liu, Yuhan Deng, Huishu Yuan, Lulin Ma

**Affiliations:** 1 Department of Radiology, Peking University Third Hospital, Beijing, China; 2 Department of Pathology, Peking University Third Hospital, Beijing, China; 3 Department of Urology, Peking University Third Hospital, Beijing, China; 4 School of Public Health, Peking University, Beijing, China

**Keywords:** Magnetic resonance imaging (MRI), Renal tumor, Inferior vena cava tumor thrombus (IVCTT)

## Abstract

**Background:**

Inferior vena cava tumor thrombus (IVCTT) invading the IVC wall majorly affects the surgical method choice and prognosis in renal tumors. Enhanced multiparameteric MRI plays an important role in preoperative evaluation. In this work, an MRI-based diagnostic model for IVCTT was established so as to guide the preoperative decisions.

**Methods:**

Preoperative MR images of 165 cases of renal tumors with IVCTT were retrospectively analyzed, and imaging indicators were analyzed, including IVCTT morphology and Mayo grade, IVCTT diameter measurements, bland thrombosis, primary MRI-based diagnosis of renal tumor, and involvement of contralateral renal vein. The indicators were analyzed based on intraoperative performance and resection scope of the IVC wall. Multivariate logistic regression analysis was used to establish the diagnostic model.

**Results:**

The morphological classification of the IVCTT, primary MRI-based diagnosis of renal tumors, maximum transverse diameter of IVCTT, and length of the bland thrombus were the main indexes predicting IVC wall invasion. The MRI-based diagnostic model established according to these indexes had good diagnostic efficiency. The prediction probability of 0.61 was set as the cutoff value. The area under the curve of the test set was 0.88, sensitivity was 0.79, specificity was 0.85, and prediction accuracy was 0.79 under the optimal cutoff value.

**Conclusion:**

The preoperative MRI-based diagnostic model could reliably predict IVC wall invasion, which is helpful for better prediction of IVC-associated surgical operations.

## Background

Renal tumors are common tumors of the urinary system and tend to invade the venous system and form tumor thrombi [1]. Approximately 4–10% of renal cell carcinomas extend to the renal vein or inferior vena cava (IVC) [2]. Tumor invasion of IVC in renal cell carcinoma (RCC) is a risk factor for recurrence and poor prognosis [3, 4]. Presently, radical nephrectomy with thrombectomy is an effective method for treating non-metastatic renal tumors with IVC tumor thrombi (IVCTTs) [5]. Furthermore, the 5-year tumor-specific survival rate can reach 50%-69% [6].

The extent of involvement of IVCTT and whether it invades the IVC wall majorly affect the surgical method choice and prognosis. If the IVCTT does not involve the IVC wall, only tumor thrombus removal is required. When the tumor thrombus invades the IVC wall, resection of the invaded IVC is necessary and even segmental resection and reconstruction of IVC vessels, which increase surgical difficulty. Therefore, preoperative judgment and prediction of IVC wall invasion are of great value for surgical planning. So AJCC TNM staging 8th edition also includes IVC wall invasion (T3c) [7]. But the current classification systems of RCC with IVCTT, such as the Mayo Clinic [8], only consider the tumor thrombus location, which is insufficient for preoperative evaluation of the possibility of IVC invasion [9]. Therefore, more accurate preoperative evaluation is also required.

Currently, magnetic resonance imaging (MRI) is the preferred examination method for diagnosing IVCTT [10], which has a high soft-tissue contrast [11] and without ionizing radiation. Furthermore, MRI is superior to computed tomography (CT) in displaying tumor thrombi and bland thrombosis in the renal vein and IVC, with high diagnostic accuracy. Although preoperative imaging of IVC invasion is not a substitute for surgical exploration, morphologic features determined using MRI may be used to predict complex IVC resection. Surgeons can use this information to optimize preoperative planning [11, 12].

In the past, MRI diagnosis of tumor thrombi and IVC wall invasion was mostly based on subjective evaluations of tumor thrombus imaging features and IVC diameter measurement, and the number of cases studied was small [11, 13, 14]. In our experience, the evaluation accuracy of a single imaging sign or diameter measurement is not adequate. Therefore, we tried to determine MRI indicators that are as objective as possible and establish a comprehensive diagnostic model of multiple indicators, to comprehensively and scientifically predict the relationship between tumor thrombi and IVC wall and intraoperative strategies.

## Materials and methods

### Clinical data

Overall, 224 hospitalized patients who underwent radical renal tumor resection and IVC tumor thrombectomy from January 2015 to December 2021 were retrospectively analyzed. The study was approved by the Ethics Committee of Peking University Third Hospital (approval no. 2018–396-01). The exclusion criteria were as follows: choice of conservative treatment willingly or owing to other reasons (*n* = 33), Mayo grade 0 thrombi (*n* = 14), incomplete or unclear preoperative MRI scans (*n* = 3), IVC compression by a renal tumor or metastatic lymph node (*n* = 3), and non-feasibility of intraoperative IVCTT removal (*n* = 6). Overall, 165 cases of renal tumors with IVCTT of Mayo grades I to IV were included. All the enrolled patients underwent preoperative enhanced MRI of the abdominal and urinary systems and radical nephrectomy and tumor embolectomy. Renal malignant tumors with IVCTT were confirmed by postoperative pathological testing.

### MRI parameters

One hundred and sixty-five patients underwent 3.0 T MRI (Discovery 750, GE, US) with an eight-channel phased-array surface coil. T2 weighted imaging (T2WI) fat suppression propeller sequence was used in axial plane. Plain T1WI was performed in axial plane, and dynamic contrast-enhanced scanning was conducted. Gadolinium DTPA (GD-DTPA, Beijing Beilu Co., Ltd.) was injected through the median vein of the anterior elbow at a dose and an injection rate of 0.2 ml/kg and 2–3 mL/s, respectively. Images were acquired at 30, 50, and 80 s after the injection. Transverse axial images in arterial, venous, and delayed phases were obtained, followed by coronal scans. The scan area included the entire IVC, with the upper margin reaching the level of the right atrium and the lower margin reaching the level of the iliac vein bifurcation. Sequence parameters were as followed Table 1.

**Table 1:** MRI Sequence parameters

	sequence	TR	TE	layer thickness	layer spacing	ETL	Flip Angle	FOV	matrix
T2WI	FS^a^ propeller	8000 ms	82 ms	5 mm	1 mm	28	110°	350 mm	320*320
T1WI	LavA-Flex	4 ms	2.4 ms	3D acquisition				360 mm	264*256
CE-MRI^**b**^	Same as T1WI								

^a^*FS* Fat suppression, ^b^*CE-MRI* Contrast-enhancement MRI

### Evaluation indicators

Images were evaluated by two radiologists with more than 6 years of experience in abdominal MRI reading. Surgical and pathological data were blinded before the evaluation, and cases were evaluated randomly. Any disagreements between the two radiologists were resolved by consulting with a third senior radiologist with 12 years of experience in abdominal MRI reading.

**Fig. 1:**
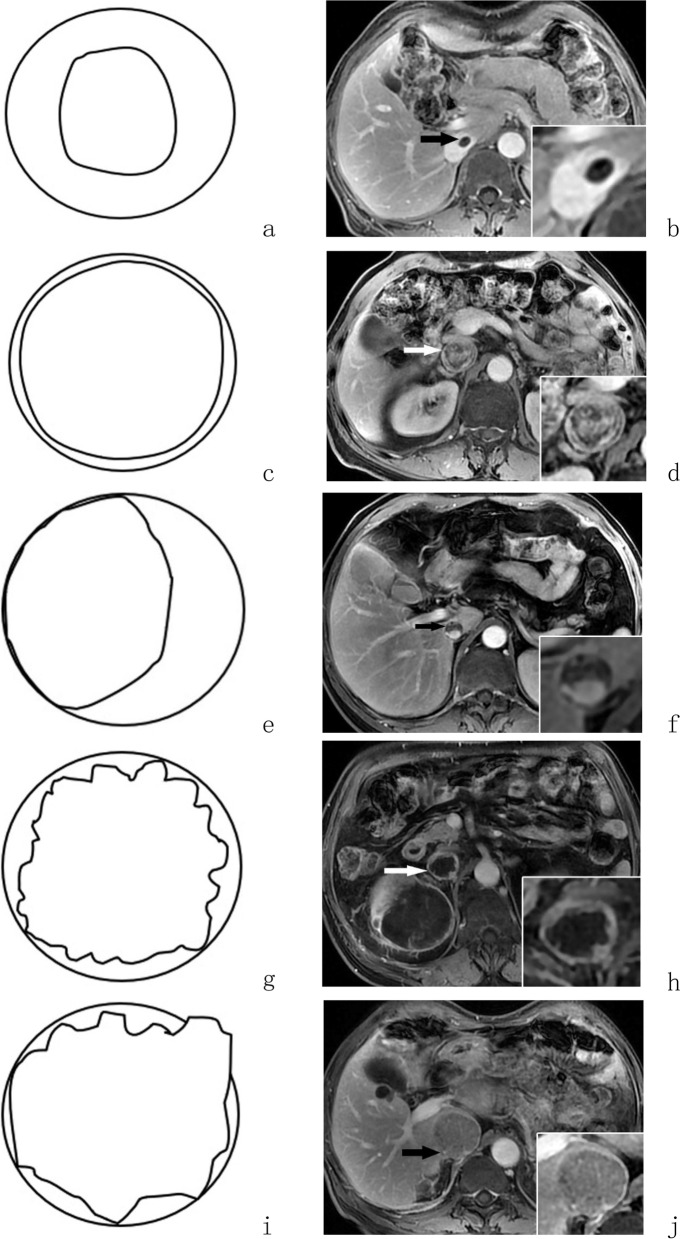
Tumor thrombus morphology types and the corresponding typical cases. Type I (**a**) and clear cell renal cell carcinoma (ccRCC) WHO/ISUP 2016 nuclear grade II (**b**), the tumor thrombus(black arrow) was small in volume and free in the inferior vena cava lumen; Type II (**c)** and ccRCC WHO/ISUP 2016 nuclear grade III (**d**), the tumor thrombus(white arrow) was plump, filling the inferior vena cava lumen, with smooth edges and a clear boundary from IVC wall, and the inferior vena cava wall is regular; Type III (**e**) and ccRCC WHO/ISUP 2016 nuclear grade I (**f**), the tumor thrombus(black arrow) did not fill the inferior vena cava lumen, but was closely related to a lateral wall; Type IV (**g**) and infiltrating upper tract urothelial carcinoma (UTUC) (**h**), the tumor thrombus(white arrow) was plump, filling the inferior vena cava lumen, with an irregular edge, and the IVC wall was irregular; Type V (**i**) and papillary renal cell carcinoma (PRCC) **(j**), the tumor thrombus(black arrow) was plump, filling the inferior vena cava lumen, with an irregular edge, and tumor thrombus protruded outwardly with obvious breach of the IVC wall

**Fig. 2:**
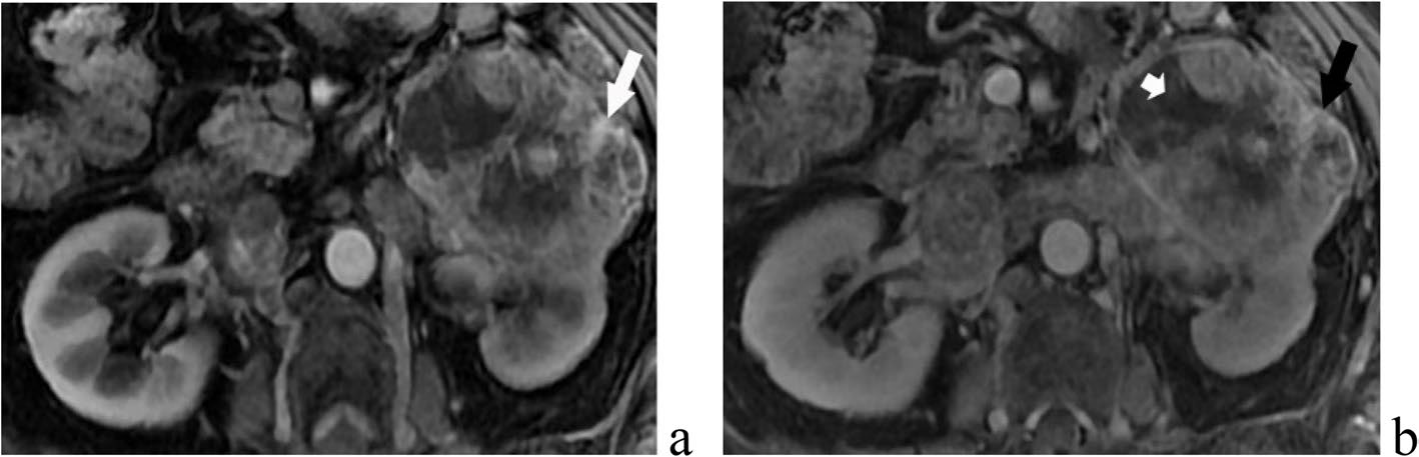
CCRCC WHO/ISUP 2016 nuclear grade I-II. The left renal mass lesion was significantly enhanced in the arterial phase (**a**, white arrow) and slightly decreased in the venous phase (**b**, black arrow), with obvious necrosis (short arrow) within the lesion and clear boundary, and there appeared to be a capsule

**Fig. 3:**
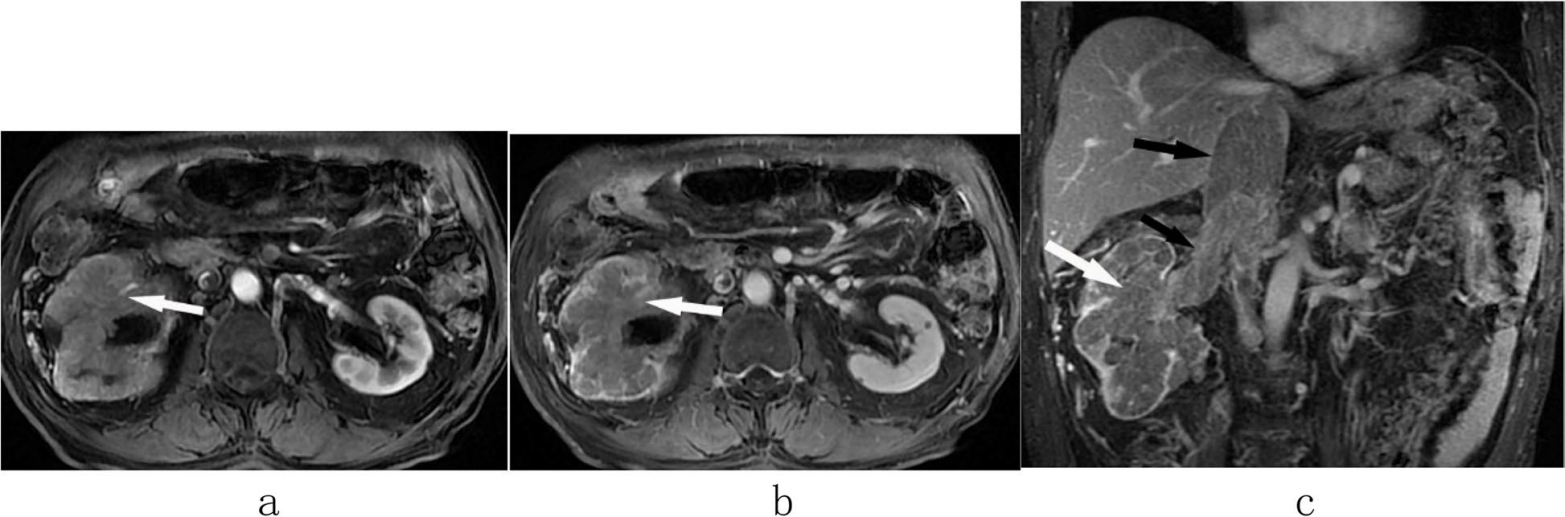
PRCC. The right renal mass lesion was mildly to moderately enhanced in the arterial phase (**a**, white arrow) and venous phase (**b**, white arrow), without clear boundary and capsule. Coronal images (**c**) showed right renal vein and IVCTT (black arrow) and right renal mass lesion(white arrow). It was the same case as Fig. 1j

**Fig. 4:**
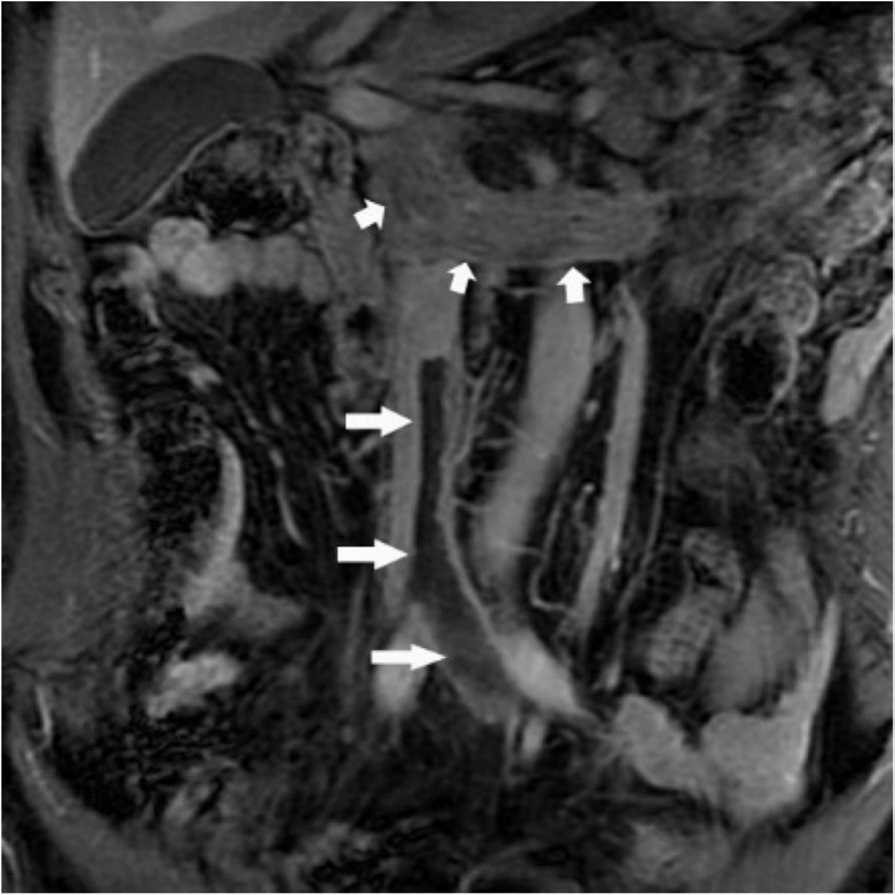
CCRCC WHO/ISUP 2016 nuclear grade III with left renal vein and inferior vena cava tumor thrombus (short arrow). A bland thrombus(long arrow) was observed at the distal end of the tumor thrombus, the lower margin reached the left common iliac vein. It was the same case as Fig. 1d

Adoption of the objective evaluation index was attempted to address issues, including IVCTT morphology and Mayo classification, preliminary MRI-based diagnosis of kidney tumors, bland thrombosis in IVC, and whether the tumor thrombus or bland thrombosis involved the contralateral renal vein. IVCTT length, maximum axis diameter of IVCTT and length of bland thrombosis were measured. The evaluation criteria for each indicator were as follows. (1) Tumor thrombus length was measured from the top to the bottom at the largest cross-section in the contrast-enhanced coronal images. (2) The maximum axis diameter of the IVCTT (IVCTTaxis) was measured at the maximum axis cross-section of the enhanced image. (3) The morphology of the IVCTT was divided into the following five types according to the axial cross-section images (Fig. 1): Type I, the tumor thrombus was small in volume and free in the IVC lumen; Type II, the tumor thrombus was plump, filling the IVC lumen but the edge of the tumor thrombus was smooth with a clear boundary from the IVC wall, and the IVC wall was regular; Type III, the tumor thrombus did not fill the IVC lumen but was closely related to a lateral wall; Type IV, the tumor thrombus was plump, filling the IVC lumen, with an irregular edge, and the IVC wall was irregular; and Type V, the tumor thrombus was plump, filling the IVC lumen, with an irregular edge, and tumor thrombus protruded outwardly with obvious breach of the IVC wall. The above five types were divided into two groups for statistical comparison. Types I–III were less likely to invade the IVC wall and were defined as group_I–III_, while the other two were more likely to invade the IVC wall and were defined as group_IV-V_. (4) Preliminary MRI-based diagnosis of the renal tumor: Preliminary classification for renal tumor diagnosis was made according to the MRI enhancement mode and tumor morphology. The preliminary diagnosis was divided into two major categories: groups A and B. The tumor showed marked contrast enhancement in arterial phase and the enhancement degree decreased in venous phase, the so-called “fast in and fast out” pattern, and the tumor was lumpy and had a clear boundary. These tumors were classified as group A (Fig. 2), while those that did not show these characteristics were defined as group B (Fig. 3). (5) The diagnostic criteria for bland thrombus were filling defect without enhancement in the IVC lumen during the contrast-enhanced scan, and the maximum length was measured from the top to bottom in coronal view image (Fig. 4).

The surgical methods and intraoperative observations for the IVCTT were recorded, which were divided into the following three types: (1) The tumor thrombus was free without adhesion to the IVC wall and could be removed directly; (2) the tumor thrombus adhered to the IVC wall or partially invaded the IVC, and thus, the invaded IVC wall needed to be removed and could be sutured or reconstructed; (3) severe IVC wall adhesion or invasion requiring segmental resection. The cases were divided into two groups: type 1 was Non-Invasive Group, which was defined as the group without IVC invasion; types 2 and 3 were Invasive Group, which was defined as the group with IVC invasion. With these two groups as a reference, imaging indicators were compared and statistically analyzed.

### Statistical analysis

The Shapiro Wilk (S-W) test was used to test whether continuous variables were normally distributed. Continuous variables conforming to normal distribution are expressed as mean ± standard deviation values; t-test was used for comparison between groups. Data that did not conform to normal distribution are represented by M(P25, P75); the Wilcoxon rank-sum test was used for comparison between groups. The categorical variables are expressed as frequencies and percentages, and χ^2^ test was used for comparison and analysis of categorical variables. When categorical variables did not meet the criteria for the χ^2^ test, Fisher’s exact probability method was used for comparison between the groups.

According to the outcome index, the data were randomly divided into training and test sets in a ratio of 8:2. The training set was used to build the model, and the test set was used to evaluate the model performance. The principal component method was used to cluster all continuous variables, and only one variable (minimum 1-R^2^) was selected from each category for multivariate logistic regression analysis. The backward method was used to screen variables and establish a prediction model. Sensitivity was plotted on the horizontal axis and (1-specificity) on the vertical axis to obtain the ROC curve to evaluate the accuracy of the prediction model for the test set. The kappa test was performed for tumor thrombus morphological typing and primary renal tumor imaging-based diagnosis to calculate the reproducibility of the evaluation by radiologists. SAS 9.4 (SAS Institute Inc., USA) was used for statistical analysis. All tests were bilateral, and *P* < 0.05 indicated statistical significance.

## Results

Among the 165 patients, 99 (60%) showed IVC wall adhesion or invasion and underwent partial or segmental IVC resection. The results of univariate analysis of imaging and surgical methods are shown in Table 2. Statistical analysis of MRI imaging indicators showed statistically significant differences between Non-Invasive Group and Invasive Group. In comparison with Non-Invasive Group, more cases with types IV and V tumor thrombus morphology showed invasion of the IVC wall than those with type I–III tumor thrombus morphology. The Mayo I IVCTT group included a higher proportion of cases showing non-invasion of IVC wall, and the Mayo II-IV IVCTT group included a higher proportion of cases showing invasion of IVC wall. The IVCTTs that invaded the vein wall was longer and thicker than those that did not. The incidence of venous wall invasion was higher in patients with bland thrombosis. The proportion of invasion to the vein wall was higher in group B renal tumor than group A. Data for the postoperative pathological types of renal tumors and corresponding changes of IVC are shown in Table 3.

**Table 2:** Single-factor analysis of the patients' basic situations and MRI indicators

		**Intraoperative performance**		
**Variable n (%)**	**Total** **(*N* = 165)**	**Group II (segmental/partial IVC excision) (*N* = 99)**	**Group I (IVC wall was not resected) (*N* = 66)**	***P***
**Sex**				0.075
Male	113(68.5)	73(73.7)	40(60.6)	
Female	52(31.5)	26(26.3)	26(39.4)	
**Age**	59.0(51.0–66.0)	59.0(50.0–66.0)	60.5(51.0–67.0)	0.751
**IVCTT morphology**				< .001
Type I–III	100(60.6)	41(41.4)	59(89.4)	
Types IV and V	65(39.4)	58(58.6)	7(10.6)	
**Mayo grade**				< .001
I	42(25.5)	9(9.1)	33(50.0)	
II	99(60.0)	71(71.7)	28(42.4)	
III	9(5.5)	8(8.1)	1(1.5)	
IV	15(9.1)	11(11.1)	4(6.1)	
**Length of IVCTT (cm)**	5.8(3.8–8.7)	6.8(5.2–9.7)	3.8(2.5–6.1)	< .001
**Maximum axis diameter (cm)**	2.9 ± 1.1	3.3 ± 1.0	2.3 ± 1.0	< .001
**Bland thrombus**				< .001
Yes	53(32.1)	46(46.5)	7(10.6)	
No	112(67.9)	53(53.5)	59(89.4)	
**Bland thrombus length**^a^ **(cm)**	0.0(0.0–2.7)	0.0(0.0–6.7)	0.0(0.0–0.0)	< .001
**Preliminary MRI-based diagnosis of renal tumors**				< .001
Group A	89(53.9)	41(41.4)	48(72.7)	
Group B	76(46.1)	58(58.6)	18(27.3)	
**Contralateral renal vein involvement**				< .001
Yes	23(13.9)	21(21.2)	2(3.0)	
No	142(86.1)	78(78.8)	64(97.0)	

The categorical variables are expressed as frequencies(percentages)

^a^Data that did not conform to normal distribution are represented by M(P25, P75), and the Wilcoxon rank-sum test was used for comparison between groups

**Table 3:** Postoperative pathological types and distribution of renal tumors

Postoperative pathologic types	primary MRI diagnosis of renal tumor		IVC wall intraoperative observations		Total
	groups A	groups B	Non-invaded	Invaded	
ccRCC I-II^a^	41	7	30	18	48
ccRCC III-IV^a^	44	17	21	40	61
PRCC	1	24	7	18	25
UTUC	0	6	0	6	6
sarcoma	0	8	2	6	8
others	3	14	6	11	17
total	89	76	66	99	165

^a^When one renal tumor contained two or more WHO/ISUP pathological grades, the highest grade was as the criterion

The Kappa test was used to test the consistency of the two radiologists' evaluation of the morphology of IVCTT and the preliminary imaging diagnosis of the renal tumor, and it showed high consistency (Kappa = 0.856, *P* = 0.300, Kappa = 0.859, *P* = 0.248, respectively).

In the univariate analysis, variables with *P* > 0.1 were eliminated. Finally, the variables included in logistic regression were as follows: sex, morphology and maximum axis diameter of the tumor thrombus, Mayo grade, length of the bland thrombus, primary MRI diagnosis of the renal tumor, and the contralateral renal vein involved. Logistic regression analysis was conducted, and four features were screened to predict the possibility of IVC wall invasion (Table 4). These variables contribute to the prediction of outcomes in the model from these statistical results. The results were as follows:

**Table 4:** Prediction model based on logistic regression

Variables	Regression coefficient	OR (95% CI)	*P*
Intercept	-1.52		0.0407
IVCTT morphology type	0.78	4.79 (1.67, 13.76)	0.0036
Primary imaging-based diagnosis of tumors (ref: group A)	0.63	3.52 (1.40, 8.89)	0.0075
Maximum axis cross-sectional diameter of the IVCTT	0.73	2.08 (1.29, 3.35)	0.0027
Length of the accompanying thrombus	0.13	1.14 (0.98, 1.33)	0.0903

$$\mathrm{Logit}(\mathrm p)=-1.52+0.78\ast(\mathrm{IVCTT\,morphology\,type})+0.63\ast(\mathrm{primary\,MRI\,diagnosis\,of\,tumor})+0.73\ast(\mathrm{maximum\,axis\,diameter\,of\,IVCTT})+0.13\ast(\mathrm{length\,of\,the\,bland\,thrombus})$$

**Fig. 5:**
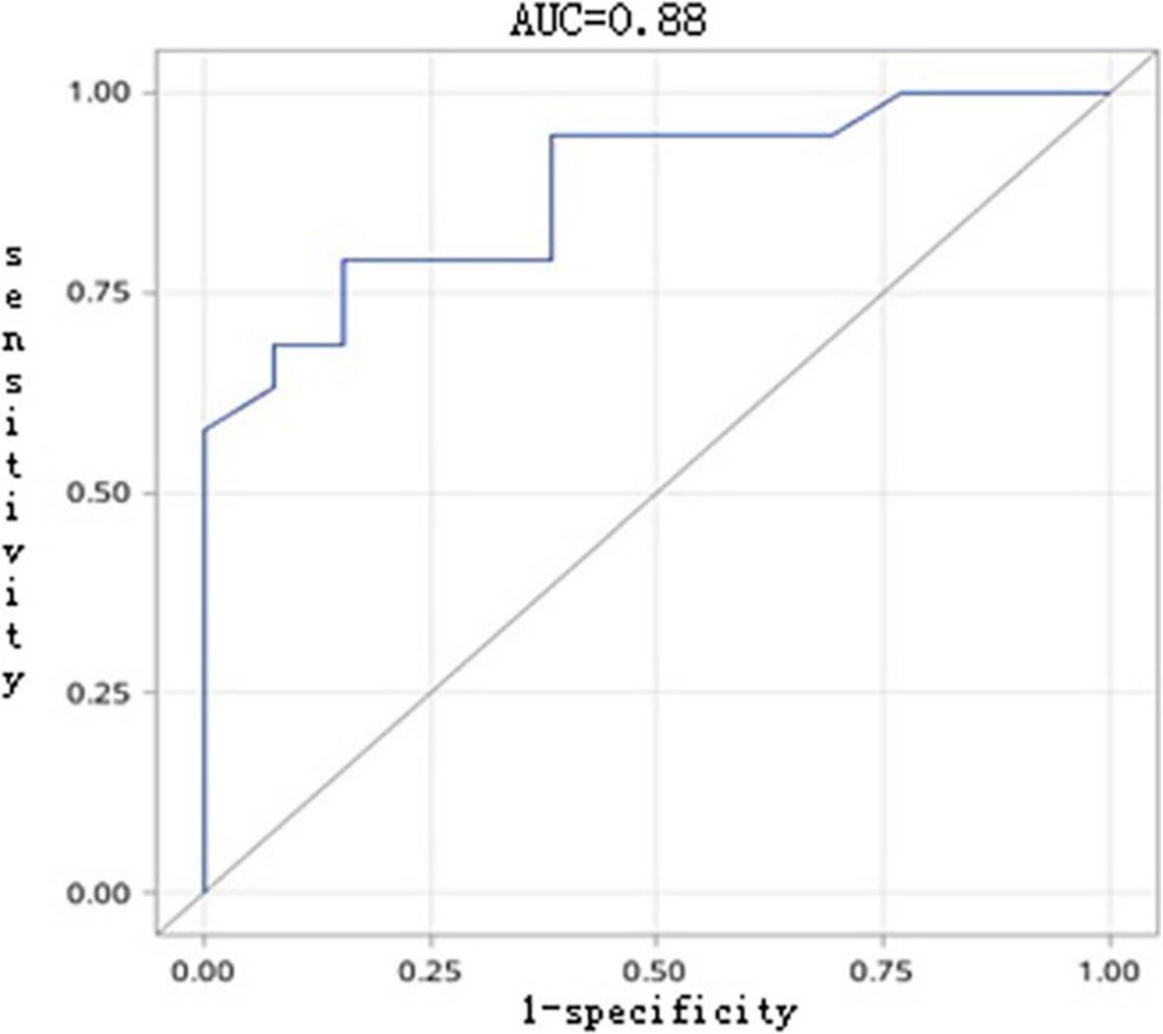
Receiver operating characteristic curve of the test set

We considered a prediction probability of 0.61 as the cutoff value, that is, if the probability value calculated from the model equation was greater than 0.61, it was judged to be positive (IVC partial or segmentalized resection seen intraoperatively), and if it is less than 0.61, it was judged to be negative (IVC was not be resected intraoperatively). The area under the curve (AUC) of the test set was 0.88 (Fig. 5). Under the optimal cutoff value, the sensitivity was 0.79, specificity was 0.85, and prediction accuracy was 0.79.

## Discussion

The basic MRI findings for IVCTT were a uniformly or non-uniformly enhanced filling defect in the IVC. At present, regular low signal of IVC wall and a clear boundary between the tumor thrombus and IVC wall is considered to indicate that there is no invasion of the vascular wall. When the volume of the tumor thrombus is large, it can be accompanied by complete obstruction or dilation of the IVC, the boundary between the tumor thrombus and the vascular wall can disappear, and the blood flow signal in the affected area will disappear. Although numerous studies have described the relationship between the IVCTT and the IVC wall, an accurate preoperative evaluation of invasion of the IVC wall by the tumor thrombus is difficult. Especially for cases in which the IVCTT completely filled the lumen, it is difficult to accurately judge the invasion of IVC wall except that the tumor thrombus obviously infringes or breaks through the IVC wall [15]. The subjective imaging characteristics of IVC wall invasion are only moderately to fairly consistent among different observers [16]. Simple contact of the IVCTT with the vascular wall is not considered a reliable predictor. Our practical experience also shows that a single index (including diameter, volume, and morphology) cannot accurately and objectively judge whether the IVC is invaded, and the signs of thickening or thinning of the IVC wall is difficult to judge accurately and reliably. Therefore, the indexes we selected were as objective as possible, and the preoperative evaluation model was established on the basis of these indexes to facilitate more scientific evaluation.

Tumor thrombus morphologic types have not been reported in the past, and are one of the important indicators of our prediction model. This index can be considered as the most important independent risk factor for IVC wall invasion in our study. Type IV and V IVCTTs are bulky and coarser, and have an irregular edge, and are suggested to show a higher possibility of IVC wall invasion. Among them, Type V classification was considered to be a reliable indicator of IVC invasion in previous studies [15]; specifically, on contrast-enhanced MR images, an enhanced thrombus signal was seen in the vascular wall and the vein wall was destroyed with the tumor thrombus breaking through the blood vessel wall, such that tumor thrombus signals were seen on both sides of the blood vessel wall. The volumes of Type I and III thrombi are mostly small, and there is a gap between them and the IVC wall, indicating that the possibility of IVC invasion is not high. Although a Type II tumor thrombus is larger and thicker, its margin is smooth, which indicates tumor thrombus expansionary growth, possibly accompanied by capsule, and a low possibility of IVC invasion. Some reports have also described the relationship between tumor thrombus morphology and prognosis. In addition to tumor size, distant metastasis and histopathological subtypes, tumor thrombus morphology can significantly affect the overall survival rate (OS) and cancer specific survival(CSS) [17]. In their study, tumor thrombus with round shape and smooth margin was defined as Type I, and was suggestive of a pseudocapsule, while tumor thrombus with irregular, sharp or friable margins was defined as Type II. The 5-year OS was 82.6% in the Type I thrombus group, and 32.5% in the Type II thrombus group [17]. Histologically, abundant fibrous tissue components were observed in pseudocapsule and the edge of the tumor thrombus, which made it more stable and reduced the risk of the embolus dislodging to distant areas before and during the operation. The morphology of the tumor thrombus shown by MR is believed to reflect its histological characteristics and the expression level of cellular adhesion molecules [18].

We found that the primary MRI diagnosis of the renal tumor was helpful in determining the invasion of the inferior vena cava wall, and was the second-most important independent risk factor for predicting IVC invasion. Tumors of group A, which mainly included low-grade clear cell renal cell carcinoma (ccRCCs), showed the so-called fast in and fast out enhancement pattern, with a lump shape and clear boundary, while tumors of group B mainly included high-grade ccRCC and other malignant tumors, such as papillary renal cell carcinoma (PRCC), sarcoma, and upper tract urothelial carcinoma (UTUC). The proportion of IVC wall invasion in group B was higher. However, due to the large tumor size and mixed signals, imaging manifestations are complicated, which increases the difficulty of preoperative qualitative diagnosis, especially for identification of various types of tumors in the group B. On the other hand, except for ccRCC, the number of cases of other types of tumor was relatively small, and more cases should be evaluated in further studies.

Our measurements for tumor embolus suggested that IVCTT with a larger transverse diameter and a longer length had a larger volume and greater probability of invading the IVC wall, consistent with the findings of previous studies. Previous studies have suggested that tumor emboli with an IVC diameter ≥ 40 mm may indicate extensive IVC wall invasion. Zini et al. [19] believed that the sensitivity of vascular wall invasion was 90% when the anterior and posterior diameter of the IVC was larger than 18 mm or the opening diameter of the renal vein was larger than 14 mm. Some scholars believe that IVC wall invasion is indicated if the IVC is completely occluded with diameter ≥ 24 mm at the level of the right renal vein [10]. Previous studies have suggested that the maximum diameter of coronal IVC is a risk factor for IVC resection [20]. We chose the maximum-axis cross-section of the tumor embolus to measure the maximum diameter of the tumor embolus, because the IVC may have a certain tilt angle in the horizontal axis, and it is more convenient to judge the distribution of the tumor embolus in the IVC lumen, so measurements in the axis plane may be more accurate.

The presence of bland thrombosis was an important predictor of IVC resection. Due to the increased immune activity and hypercoagulability of patients with IVCTT, blood is prone to stasis in the vein, and simultaneous damage to the vascular endothelium and activation of coagulation factors may cause the formation of bland thrombus with tumor thrombus [21, 22]. This combination can increase the difficulty of surgery; moreover, thrombus near the heart may be unstable and easy to dislodge, causing pulmonary embolism; Thrombosis exists at the distal end of the tumor thrombus, which is easy to cause complete obstruction of the IVC and form collateral circulation. Large veins with compensatory hyperplasia will appear around the tumor, increasing the risk of intraoperative bleeding. Therefore, whether the IVCTT is combined with thrombus is of great significance for the selection of surgical methods. The MR imaging feature of thrombus was mainly unenhanced filling defect in IVC that extended along the vessel and was often located at the distal end of the tumor thrombus. In this group, 32.1% (53/165) of the cases showed a thrombus distal to the tumor thrombus, and the proportion of IVC resection was very high (46/53, 86.8%).

Our data indicated that 60% (99/165) of the cases had IVC invasion. Using univariate and multivariate analyses, we found four strong indicators and significant risk factors for IVC invasion, namely, tumor thrombus morphology, primary MRI diagnosis of the tumor, maximum axis diameter of tumor thrombus, and length of the bland thrombus. An MR diagnostic model was proposed with good diagnostic efficiency and an AUC of 0.88, which showed good predictive value. Therefore, this model may be used for preoperative planning and individualized calculation of resection probability for each case, and it can also provide the possibility of one-stop preoperative diagnosis.

This study had some limitations: (1) It was a retrospective study, and there may be potential selection bias in the tumor thrombus Mayo grade and tumor pathological type. (2) The reference criteria for venous wall invasion used intraoperative findings without using pathological assessments. However, the use of pathological assessments as a standard for invasion of the IVC wall has certain limitations, because the location of the IVC without resection cannot be evaluated for invasion, nor can it reflect the surgical decision during surgery. (3) This study is limited to the clinical experience and practice of a single institution, and multi-center validation is needed in the future, in addition to evaluation of more cases to verify the effectiveness of the model. (4) Radiomics methods were not used. But radiomics methods require additional ROI delineation and post-processing, and without uniform standard for different institutions and MRI instruments until now. Our The diagnostic model is relatively simple and reliable and applicable to different MRI instruments. (5) The male population accounts for the large majority of studied subjects (68.5%), and this implies that the results are more representative of the male population. Further studies with more female population are needed in the future.

## Conclusion

We build the preoperative MRI-based diagnostic model, which can reliably predict IVC wall invasion, and is helpful for better preoperative decision of IVC-associated surgical operations. The indices, including tumor thrombus morphology, primary MRI diagnosis of the tumor, maximum axis diameter of tumor thrombus, and length of the bland thrombus, are very important contents for GU Radiologists reporting these cases.

## Data Availability

The datasets analyzed during the current study are available from the corresponding author on reasonable request pending the approval of the institution(s) and trial/study investigators who contributed to the dataset.

## Abbreviations

MRI: Magnetic resonance imaging
IVC: Inferior vena cava
IVCTT: Inferior vena cava tumor thrombus
RCC: Renal cell carcinoma
ccRCCs: Clear cell renal cell carcinoma
PRCC: Papillary renal cell carcinoma
UTUC: Upper tract urothelial carcinoma
IVCTTaxis: The maximum axis diameter of the IVCTT
OS: Overall survival rate
CSS: Cancer specific survival
